# A long term bone and renal safety of TAF treatment on renal transplant recipients

**DOI:** 10.1016/j.bj.2025.100833

**Published:** 2025-02-15

**Authors:** Desmond Y.H. Yap, Cheng-Kun Wu, Colin Tang, Kuo-Chin Chang, Wen-Chin Lee, David T.W. Lui, Maggie K.M. Ma, Tsung-Hui Hu, Tak Mao Chan

**Affiliations:** a Division of Nephrology, Department of Medicine, School of Clinical Medicine, Queen Mary Hospital, The University of Hong Kong, Hong Kong; b Division of Hepato-Gastroenterology, Department of Internal Medicine, Kaohsiung Chang Gung Memorial Hospital and Chang Gung College of Medicine, Taiwan; c Division of Nephrology, Department of Internal Medicine, Kaohsiung Chang Gung Memorial Hospital and Chang Gung College of Medicine, Taiwan; d Division of Endocrinology and Metabolism, Department of Medicine, School of Clinical Medicine, LKS Faculty of Medicine, The University of Hong Kong, Hong Kong

**Keywords:** Tenofovir alafenamide, Hepatitis B, Kidney transplantation, Osteoporosis

## Abstract

**Rationale & objective:**

The data on tenofovir alafenamide (TAF) in kidney transplant recipients (KTRs) with chronic hepatitis B virus (HBV) infection is limited.

**Study design:**

Retrospective cohort study.

**Setting & study populations:**

HBsAg-positive KTRs who received TAF between 2019 and 2022 were included in the analysis, categorized into treatment-naïve and treatment-experienced groups. Additionally, a subgroup of patients receiving ETV was analyzed for comparison.

**Results:**

Four treatment-naïve (Group I) and 35 treatment-experienced (Group II) patients received TAF for 26.4 ± 11.3 and 43.7 ± 19.0 months, respectively. Both groups showed significant HBV DNA reduction, but Group I achieved higher rates of undetectable HBV DNA (50%, 75%, 75%, 100% at 6, 12, 24, 30 months, compared with 16.7%, 25.3%, 31.4%, 34.7% in Group II, *p* = 0.018). Renal allograft function remained stable during follow-up, and bone toxicity was minimal. For ETV, 10 patients demonstrated excellent viral suppression (HBV DNA undetectable in 70% at 48 weeks and 100% at 96 weeks) with stable renal function over a median of 5.2 years.

**Limitations:**

Retrospective study with a lack of prospective comparison of TAF and ETV.

**Conclusions:**

Our results suggest that TAF provides favorable efficacy, renal safety, and tolerability in KTRs. ETV also provided effective and sustainable viral suppression. TAF may offer additional advantages such as no concern of viral resistance and dose adjustment by eGFR levels for long-term management of HBsAg-positive KTRs.

## Highlights

• TAF is effective and well-tolerated in KTRs with HBV, showing stable renal function and minimal bone toxicity over 42 months.
• Both TAF and ETV provide effective viral suppression, but TAF requires no dose adjustment based on eGFR and has no resistance.
• Unlike TDF, TAF has no significant renal toxicity or bone loss, making it ideal for KTRs on long-term steroids and immunosuppression.

## 1 Introduction

Chronic hepatitis B virus (HBV) infection is associated with various short- and long-term hepatic complications and hence excess mortalities in kidney transplant recipients (KTRs) [[1], [2], [3], [4], [5]]. The use of oral nucleotide/side analogues (NA) in KTRs has significantly improved patient outcomes, but the earlier generation of NAs, such as lamivudine (LAM) was associated with high rates of drug resistance after prolonged administration [[6], [7], [8], [9]]. Our group and their investigators have previously reported the short- and long-term efficacy and tolerability of entecavir (ETV) in HBsAg + KTRs [[10], [11], [12]], and the current guidelines have also recommended ETV as first-line NA for treatment-naïve HBsAg + KTRs [13]. However, ETV resistance is not uncommon among HBsAg + KTRs receiving chronic immunosuppression, especially among those with prior LAM-resistance [14]. In this context, adefovir dipivoxil (ADV) or tenofovir disoproxil fumarate (TDF) has been used as salvage therapy for LAM- or ETV-resistance, but these nucleotide analogues may cause renal allograft dysfunction that require drug discontinuation in up to 30–50% of KTRs [[15], [16], [17], [18], [19]]. Reduction in bone mineral density (BMD) are also important considerations for KTRs receiving long-term corticosteroids and TDF treatment [[20], [21], [22], [23]].

Tenofovir alafenamide (TAF) is a pro-drug of tenofovir and is licensed for the treatment of human immunodeficiency virus (HIV) and chronic HBV infection. TAF was developed with the goal of reducing the long-term toxicities of TDF while maintaining its advantages on drug efficacy and barrier to resistance. Studies in the general population suggest that TAF has potent activity against both treatment-naïve and drug-resistant HBV infection [[20], [21], [22]], and shows very low resistance rates even with long-term treatment [24,25]. Due to its distinct pharmacological properties, TAF shows a much lower risk of renal and bone toxicities when compared to TDF [[20], [21], [22]]. These properties have rendered TAF an attractive option for the long-term management of HBsAg + KTRs. However, data on its efficacy and safety in HBsAg + KTRs is limited, and therefore, our current study aimed to report the efficacy, tolerability and clinical outcomes of HBsAg + KTRs treated with TAF. Additionally, we compared treatment-naive and treatment-experienced patients to assess any differences between the two groups.

## 2 Patients and methods

### 2.1 Patients

We retrospectively reviewed case records of all deceased- or live-donor KTRs who were followed at the Nephrology unit of Queen Mary Hospital, Hong Kong and Chang Gung Memorial Hospital, Taiwan, during the period of 2019–2022 (TAF was available in both centres since January 2019). The follow-up period is extended until December 2022. KTRs who were HBsAg-positive and had received TAF were included for analysis. Patients who tested positive for anti-HCV and/or HCV RNA or had significant alcohol consumption were excluded. The study was approved by the Institutional Review Board of the Chang Gung Medical Foundation in Taiwan (IRB No.: 202301008B0) and the University of Hong Kong/Hospital Authority Hong Kong West Cluster (HKU/HA HKW IRB). Patients who continuously used entecavir (ETV) were also shortly reviewed.

### 2.2 Immunosuppressive regimen and follow-up protocol

According to the local healthcare policies, HBsAg-positive patients receive renal allografts from HBsAg-positive donors. Standard prophylactic immunosuppressive treatments for KTRs in both centres were a triple regimen comprising prednisolone, calcineurin inhibitors [either cyclosporine A (CYA) or tacrolimus (TAC)], and mycophenolate mofetil (MMF). The immunosuppressive regimen could be adjusted during follow-up, taking into considerations any severe uncontrolled sepsis, treatment intolerance, and development of malignancy.

Patients were seen every 2–4 weeks for the first 6 months, then every 4–6 weeks for the subsequent 6 months, and every 8–12 weeks after the first year. During each follow-up, renal and liver biochemistry, estimated glomerular filtration rates (eGFR), blood phosphate (PO4) levels, 12-h trough CYA or TAC levels, urine protein-to-creatinine ratios and clinically significant events were monitored [26]. Circulating HBV DNA was measured with quantitative real-time polymerase chain reaction (qPCR), which had a sensitivity of 100 copies/mL (5 copies/mL = 1 IU/mL) [27], at every 6 months. Virological breakthrough was defined as resurgence of HBV DNA >500 IU/mL after initial successful suppression of HBV DNA. Alpha-feto protein (AFP) was monitored half-yearly. Annual ultrasound surveillance was performed to detect cirrhosis and hepatocellular carcinoma (HCC). Liver stiffness was assessed by elastography (Fibroscan, Echosens, Paris, France) in selected patients, and hepatic cirrhosis was denoted as liver stiffness measurement >9.0 kPa in patients with normal ALT level or >12.0 kPa in patients with ALT 1–5-fold the upper limit of normal [28]. Dual-energy X-ray absorptiometry (DXA) was performed before TAF treatment and also at 6–12 months after TAF treatment to evaluate bone changes. To detect more subtle changes in renal allograft function, we also measured kidney injury markers, including KIM-1 and IL-18 using commercially available ELISA kits (KIM-1 - Quantikine®, R&D Systems China, Shanghai, China PRC; IL-18 - MBL™, MA, USA) before and after TAF treatment.

### 2.3 Anti-viral treatment

In treatment-naïve KTRs, TAF was initiated at 25 mg daily when HBV DNA level was ≥5 × 10^3^ IU/mL with normal ALT or 5-5x10^3^ IU/mL with abnormal ALT. TAF was also commenced at 25 mg daily in patients who had prior exposure to other NAs but showed incomplete viral suppression (HBV DNA ≥5 × 10^3^ IU/mL) or virological breakthrough (reappearance of HBV DNA >500 IU/mL after initial successful suppression). Regarding treatment-experienced KTRs, 11 patients were lamivudine experienced, 2 were adefovir experienced, 4 were telbivudine experienced, 12 were entecavir experienced, 15 were tenofovir dixoproxil fumarate (TDF) experienced, and were subsequently switched to tenofovir alafenamide (TAF) therapy after 2019.

### 2.4 Statistical analysis

Continuous variables were expressed as mean ± S.D., and analyzed with Student's *t-test* or Mann-Whitney test where appropriate. Categorical variables were expressed as frequency (percentage), and analyzed with Chi-square test or Fisher-Exact test where appropriate. The patient and graft survival were estimated by Kaplan-Meier methods and survivals were compared by log-rank test. All statistical analyses were performed by statistical software SPSS (Version 24, IBM), and a p-value <0.05 was considered statistically significant.

## 3 Results

### 3.1 Patients

Thirty-nine HBsAg-positive KTRs received TAF for 42.0 ± 19.0 months. Four patients were treatment-naïve, while 35 patients had received other NAs after kidney transplantation prior to initiation of TAF (treatment-experienced group) [Table 1]. The duration of TAF therapy for treatment-naïve and treatment-experienced patients were 26.4 ± 11.3 months and 43.7 ± 19.0 months, respectively.

**Table 1:** Clinical characteristics of 39 HBsAg-positive kidney transplant recipients who have received tenofovir alafenamide (TAF) treatment.

	Treatment-naïve (n = 4)	Treatment-experienced (n = 35)	P-values
**Age (years)**	51.7 ± 10.5	57.5 ± 9.6	0.359
**Sex (Male/Female)**	1/3	25/10	0.099
**Mode of dialysis before transplantation** (HD/PD)	4/0	30/5	0.418
**Type of transplant** (deceased-/live-donor)	4/0	29/6	0.368

**Duration of follow-up after transplantation (years)**	8.4 ± 8.9	17.4 ± 7.8	0.134
**Duration of TAF treatment (months)**	26.4 ± 11.3	43.7 ± 19.0	0.085

**History of hepatic complications before initiation of TAF**			
Cirrhosis	1	3	0.363
Hepatocellular carcinoma	0	2	0.624

**Laboratory parameters upon initiation of TAF**			
HBV DNA (IU/mL)	9.6 × 10^6^±1.7+10^7^	2.9 × 10^7^±1.7 × 10^8^	0.008
ALT level (U/L)	54.5±-59.0	24.0 ± 13.9	0.195
HBeAg Status (+ve/-ve)	2/2	5/30	0.141
Serum Cr level (μmol/L)	120 ± 21.8	152±-83.5	0.515
eGFR (mL/min)	43.6 ± 11.9	48.2 ± 20.9	0.762

Abbreviations: ALT: alanine transferase; AZA: azathioprine; CNI: calcineurin inhibitors; Cr: creatinine; eGFR: estimated glomerular filtration rate; TAF:Tenofovir; HBV: hepatitis B virus; HD: hemodialysis; mTOR inhibitor: mammalian target of rapamycin inhibitors; MMF: mycophenolate mofetil; PD: peritoneal dialysis.

### 3.2 Virological response

For the treatment-naïve group, the mean HBV DNA was 9.6 × 10^6^±1.7 × 10^7^ IU/mL before initiation of TAF. They began using the treatment in December 2019, September 2020, November 2020, and January 2021, respectively. The HBV DNA became undetectable after 6.0 ± 5.2 months. Effective HBV DNA suppression was sustained for up to 24 months. For the treatment-experienced group, mean HBV DNA was 2.9 × 10^7^±1.7 × 10^8^ IU/mL before commencement of TAF and dropped to 1.1 × 10^3^±3.9 × 10^3^ IU/mL, 30.9 ± 65.8 IU/mL, 5.0 ± 12.2 IU/mL, 2.0 ± 6.9 IU/mL, and eventually became undetectable after 6, 12, 24, 36, 42 months respectively. Treatment-naïve patients showed higher rates of HBV undetectability over time (50%, 50%, 75%, 75%, 100% at 3, 6, 12, 24, and 30 months after treatment respectively, compared with 5.6%, 16.7%, 25.3%, 31.4%, 34.7% respectively in treatment-experienced patients, *p* = 0.018). The treatment-naïve group also achieved earlier HBV undetectability after 6.0 ± 5.2 months (compared with 13.8 ± 9.6 months in the treatment-experienced group, *p* = 0.106).

**Fig. 1:**
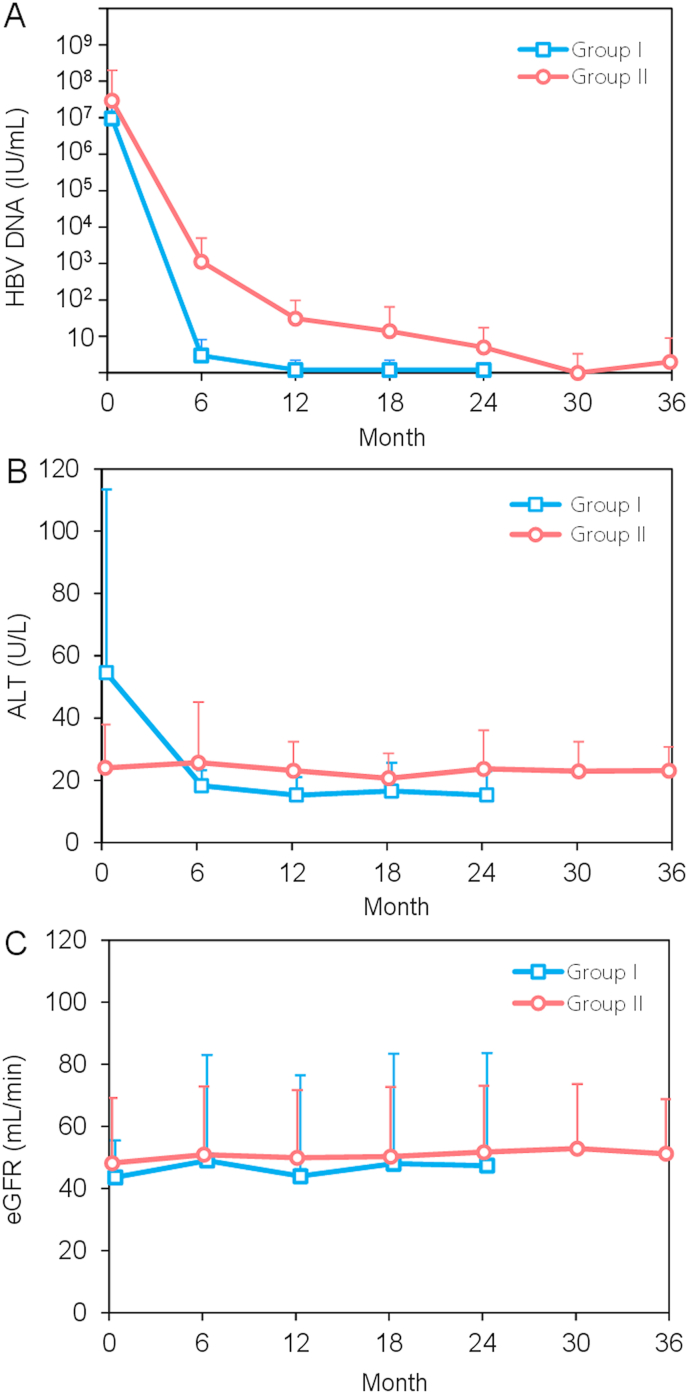
Longitudinal changes in (A) HBV DNA (B) ALT levels and (C) renal allograft function in treatment-naïve (Group I0 or treatment-experienced (Group II) HBsAg-positive kidney transplant recipients.

In the treatment-naïve group, mean ALT was 54.5 ± 59.0 U/L before the commencement of TAF, and dropped to 18.3 ± 4.9 U/L, 15.3 ± 4.8 U/L and 15.3 ± 7.6 U/L after 6, 12, and 24 months. The mean time-to-ALT normalization was 4.5 ± 2.1 months in the treatment-naïve group. For the treatment-experienced group, mean ALT in treatment-experienced group was within the normal limits throughout the study period (24.0 ± 13.9 U/L at baseline vs 23.1 ± 7.6 U/L at 36 months, *p* = 0.724) [Fig. 1 B]. Two patients in the treatment-naïve group and five patients in the treatment-experienced group were HBeAg positive, and e-seroconversion occurred in 2 patients (both in the treatment-experienced group) who were HBeAg-positive before TAF treatment. One patient (2.6%) developed a virological breakthrough at 21 months, following a period of effective HBV DNA suppression. This patient had been treated with LAM and telbivudine before switching to TAF. The mean HBV DNA and ALT at the time of the virological breakthrough were 3.6 × 10^3^ IU/mL and 14 U/L, respectively.

### 3.3 Patient survival and hepatic complications

The overall patient survival was 96.7 % at 3 years. One patient died because of septic shock in this cohort (unrelated to liver complications). There was no difference in patient survival between the two groups (100% and 96.6% in treatment-naïve and treatment-experienced patients respectively, *p* = 0.853). One patient (2.6%) in the treatment-experienced group developed liver cirrhosis at 25 months after TAF. There was no difference in the baseline HBV DNA level between patients with or without hepatic complications (22 IU/mL vs. 24.1 IU/mL).

### 3.4 Impact on renal allograft function

Four patients developed renal allograft failure (one in the treatment-naïve group after 3 months, and three in the treatment-experienced group after 12.5 ± 9.0 months). The mean eGFR at the time of TAF initiation was 18.0 ± 12.5 ml/min in those with graft failure. The overall graft survival was 89.4%. The mean eGFR was 43.6 ± 11.9 mL/min for the whole group before initiation of TAF and was 47.4 ± 36.2 after 2 years of treatment (*p* = 0.493, compared with baseline). There was no significant difference in the slope of eGFR change before and after TAF treatment (−0.5 ± 6.6 mL/min/year vs. −0.2 ± 7.2 mL/min/year; *p* = 0.937). The levels of KIM-1 and IL-18 were similar before and after TAF treatment (112.6 ± 76.5 vs. 172.0 ± 134.8 and 255.9 ± 54.0 vs. 303.4 ± 152.4 respectively, *p* > 0.05 for all compared with baseline).

**Fig. 2:**
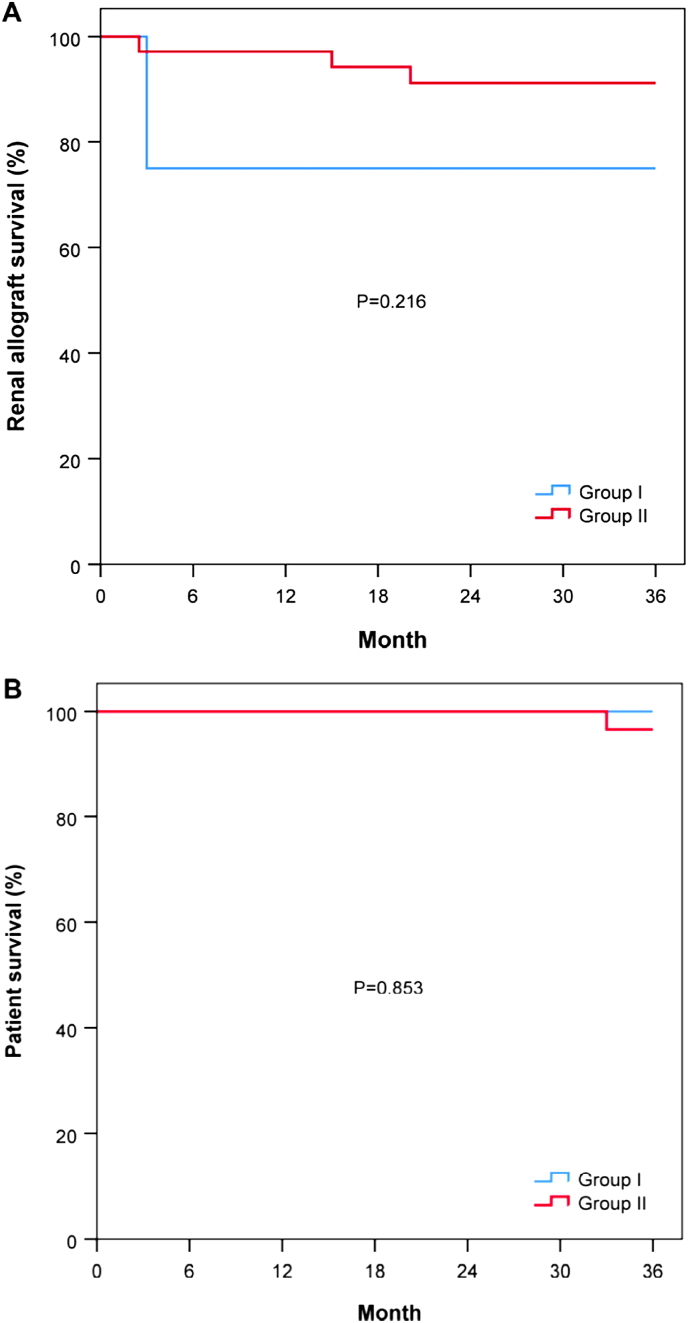
(A) Renal allograft and (B) patient survival in 39 HBsAg-positive kidney transplant recipients who have received tenofovir alafenamide (Group I - Treatment-naïve; Group II – Treatment-experienced).

As shown in [Fig. 2], the renal allograft graft survival was similar between the two groups (75.0 % vs. 91.2 % at 3 years in treatment-naïve and -experienced patients respectively, *p* = 0.216). The eGFR before initiation of TAF and after 2 years of treatment showed no significant difference in both groups (43.6 ± 11.9 mL/min vs. 47.4 ± 36.2 mL/min in the treatment-naïve group, *p* = 0.863; and 48.2 ± 20.9 ml/min vs. 52.9 ± 20.7 min/mL for the treatment-experienced group, *p* = 0.635) [Fig. 1 C].

### 3.5 Changes in serum phosphate levels

The baseline PO4 level before initiation of TAF was 1.1 ± 0.3 mmol/L. The mean PO4 levels at 3, 6, 12, 24, and 36 months after TAF treatment were 1.1 ± 0.3 mmol/L, 1.1 ± 0.3 mmol/L, 1.1 ± 0.3 mmol/L, 1.0 ± 0.3 mmol/L and 1.0 ± 0.2 mmol/L respectively (*p* = 0.369, compared with baseline). Five patients (12.8%) developed hypophosphatemia (PO4 <0.88 mmol/L) at 17.4 ± 10.5 months.

### 3.6 Changes in bone mineral density

**Fig. 3:**
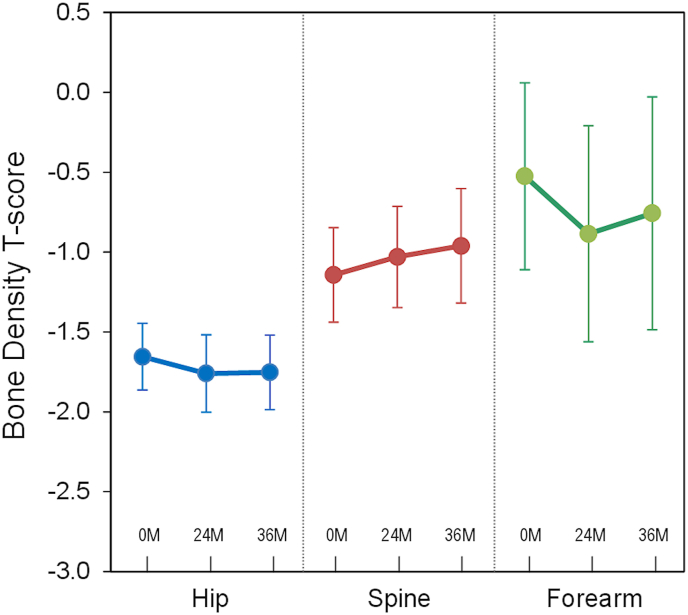
Changes in bone density (T-score) before and after treatment with tenofovir alenfenamide in HBsAg + kidney transplant recipients.

The baseline T-score of hip, spine, and forearm were −1.7 ± 0.9, −1.1 ± 1.3 and −0.5 ± 1.9, and were −1.8 ± 1.0, −1.0 ± 1.5, and −0.8 ± 2.3 respectively after 36 months of TAF treatment (*p* = 0.303, 0.242 and 0.735, compared with baseline) [Fig. 3]. Three patients (7.7%) had new-onset osteopenia at 18, 20 and 24 months respectively. One patient (2.6%) developed new-onset osteoporosis at 24 months.

### 3.7 Entecavir cohorts

**Fig. 4:**
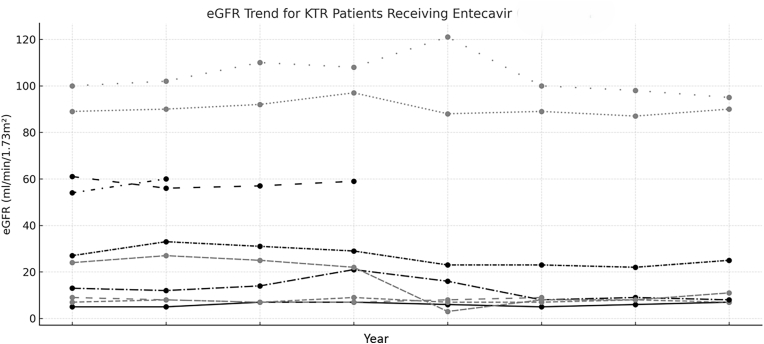
eGFR trends for KTR patients receiving entecavir in the first 3 years.

Finally, we further analyzed 10 patients who continued using ETV until now due to physicians’ preferences. The period of treatment ranged from 3.6 to 6.8 years, with a median of 5.2 years. These patients exhibited excellent viral suppression to undetectable HBV DNA 70% at 48 weeks, 100% at 96 weeks, and 100% at 144 weeks. Additionally, as shown in [Fig. 4], their renal function (eGFR) remained stable throughout the first three years, with no significant changes observed (*p* > 0.05). However, there was limited data of sequential change of bone density for comparison in these patients. There is no difference of viral suppression as well as eGFR evolution when compared ETV with TAF cohort in three years.

## 4 Discussion

Tenofovir alafenamide has been shown to effectively suppress the hepatitis B virus. However, few studies have explored the efficacy and safety of tenofovir alafenamide in KTRs who also have hepatitis B. For treatment-naïve HBsAg-positive KTRs, this study found that TAF therapy was associated with a rapid reduction in HBV DNA levels, with the majority of patients achieving undetectable viral levels after 6 months of treatment. The decrease in HBV DNA levels was also accompanied by an improvement in ALT levels. Although virological response was relatively slower in the treatment-experienced group, most patients eventually achieved undetectable HBV DNA levels by around one year and the liver transaminase levels were within normal limits throughout the treatment period. In addition to excellent viral suppression, there are also benefits from TAF, such as no resistance in long-term follow-up, no need of dosage adjustment to the change of renal function and hepatic impairment, as well as no concern of food intake when compared with other antiviral agents. These observations suggested the good clinical efficacy of TAF in both treatment-naïve and treatment-experienced HBsAg + KTRs, and were in line with that in the general population [[20], [21], [22]]. Of note, the patients who developed virological breakthrough had previous exposure to LAM and telbivudine before switching to TAF. As in the general population, TAF treatment was also associated with very low resistance rates in HBsAg + KTRs, and this is clinically important because KTRs generally require life-long immunosuppression to prevent organ rejection and prolonged NAs treatment [24,25].

A previous study reported that ETV was effective in treating HBsAg + KTRs [11], either with treatment-naïve or treatment-experienced status. As for ETV, our earlier study conducted from 2007 to 2011 demonstrated that entecavir (ETV) treatment in KTRs with chronic hepatitis B had minimal impact on renal function [11]. The glomerular filtration rate (GFR) remained stable throughout the treatment period, with no significant renal graft dysfunction observed. Furthermore, there were no reports of lactic acidosis, myopathy, or other serious adverse effects. These findings indicate that ETV is a safe and effective antiviral therapy for managing chronic hepatitis B in KTRs without compromising kidney function. However, concerns remain regarding the use of ETV in renal transplant recipients. Dose adjustments are required based on eGFR levels, and there is a possibility of resistance (albeit low) after long-term therapy, especially when compared with TAF. Consequently, most patients in the present study are now receiving TAF.

The HBV DNA levels became undetectable for 96% after 52 weeks of ETV treatment without viral resistance. In this current study, 10 patients in Taiwan were switched from ETV to TAF and similarly demonstrated effective viral suppression. In addition, 1 patient on telbivudine and 15 patients on TDF were subsequently switched to TAF therapy. Participants eligible for the switch to TAF may have done so due to concerns about achieving a virological or biochemical response, treatment adherence, or safety issues associated with the nucleos(t)ide analog used for treatment. Overall, no HBV reactivation occurred in any participant who switched to TAF.

In our cohort, no patient died of liver-related complications and only one developed cirrhosis, insinuating the efficacy of TAF in preventing long-term hepatic complications. One should appreciate that most patients in this study were treatment-experienced, and hence, it remains unknown whether the viral suppressive effect of prior NAs treatment may have also contributed to the very low risk of hepatic sequelae. Notwithstanding, previous studies in general population demonstrated that TAF treatment in patients with chronic HBV infection was associated with lower risk of liver complications such as HCC compared to other NAs, possibly related to more effective viral suppression [29].

One major concern of using nucleotide analogues (e.g. ADV or TDF) in HBsAg + KTRs is nephrotoxicity. Previous small series showed that up to 30–50% of HBsAg + KTRs treated with ADV or TDF might require treatment discontinuation as a result of allograft dysfunction [[15], [16], [17], [18], [19]]. Our current data indicated that TAF treatment in HBsAg + KTRs did not cause deterioration in renal allograft function and the overall renal allograft survival was rather favorable. Notably, the eGFR had remained relatively stable during the study period of approximately 3 years, with no significant difference between the eGFR slopes before and after TAF treatment. The insignificant changes in kidney injury markers (KIM-1 and IL-18) before and after TAF treatment further supported its renal safety in HBsAg + KTRs. Indeed, previous studies in the general population have demonstrated that TAF was associated with a much lower risk of nephrotoxicity compared with TDF [[20], [21], [22]]. Other studies in liver transplant recipients also reported the renal benefits of TAF compared to TDF [30,31]. Putative mechanisms for the lower risk of nephrotoxicity include less uptake of TAF into renal tubular cells because it is not is a substrate of renal organic anion transporter and also its lower circulating concentration due to its better uptake into hepatocytes [[32], [33], [34]]. Nonetheless, four patients developed renal allograft failure - three occurred at approximately 12 months after TAF treatment. These four patients all had significant allograft dysfunction at the time of TAF initiation, and therefore it remained unclear whether these allograft failures were the result of progressive graft CKD or they might be contributed by TAF therapy. Our findings also highlight the careful selection of HBsAg + KTRs for TAF treatment and close monitoring of allograft function after the commencement of therapy. Hypophosphatasemia is also a recognized renal complication of TAF treatment. In this study, we observed a relatively low risk of hypophosphatemia compared to previous studies in patients without significant renal impairment [[20], [21], [22],^35^]. While we postulate that TAF may cause less renal tubular defects, it remains possible that the lower incidence of hypophosphatemia might be related to impaired phosphate clearance in KTRs with chronic allograft dysfunction.

Another important issue of administering tenofovir derivatives in HBsAg + group is the effect on bone mineral density. Previous studies have demonstrated a substantial enhancement in bone mineral density and a reduction in the risk of osteoporotic fractures among individuals undergoing TAF treatment, as compared to those receiving TDF treatment [36,37]. This issue is particularly critical in organ transplant recipients, as they require long-term administration of immunosuppressive agents and potential corticosteroid use. However, the majority of the literature predominantly focuses on the safety of renal function. This article represents the first study targeting kidney transplant recipients to examine the impact of TAF treatment on changes in bone mineral density. Consistent with data in the general population, our results showed that TAF did not cause excessive osteopenia or osteoporosis in KTRs [[20], [21], [22], [23]]. The changes in the T-score at the femur, forearm, and lumbar spine before and after TAF therapy were also insignificant. The use of TAF does not result in additional bone loss in HBsAg-positive kidney transplant recipients. Given these findings, there is now a general recommendation to replace TDF with TAF in HBsAg-positive kidney transplant recipients. TAF offers superior renal and bone safety profiles, along with comparable or better antiviral efficacy, as well as no concern of dose adjustment by eGFR levels, making it a preferred option for long-term management in this vulnerable patient population.

One key limitation of this study is that there are no prospective comparative groups (e.g. TDF or ETV) to better demonstrate the efficacy and renal safety of TAF in this study. However, the number of KTRs with chronic HBV infection is relatively small and assign patients to different NA treatments. Furthermore, many patients in this cohort had prior exposures to other NAs, and there was no mutational analysis for patients who developed virological breakthroughs after TAF therefore resistance to TAF could not be confirmed. Nevertheless, our series was by far the largest cohort on the use of TAF in HBsAg + KTRs and represented the real-world experience on the efficacy and safety of TAF in this vulnerable patient subgroup. Such data substantiate the use of TAF in the management of KTRs with chronic HBV infection, especially those who have previously received other NAs or had anti-viral resistance.

## Conclusion

TAF treatment was associated with favorable efficacy as well as renal and bone safety in HBsAg + KTRs.

## Transparency declarations

None to declare.

## Author contribution roles

Desmond Y. H. YAP: conceptualization, data curation, formal Analysis, writing (original draft, review, and editing).

Cheng-Kun Wu: data curation, review, and editing.

Colin TANG: formal analysis.

Kuo-Chin Chang, Wen-Chin Lee, David T. W. LUI, Maggie K. M. MA: data curation.

Tsung-Hui, HU, Tak Mao CHAN: conceptualization, supervision, writing (original draft, review and editing).

## Funding

This study was supported by a research grant from CMRPG8N1011.
